# The protein disulfide isomerase 1 of *Phytophthora parasitica* (PpPDI1) is associated with the haustoria-like structures and contributes to plant infection

**DOI:** 10.3389/fpls.2015.00632

**Published:** 2015-08-18

**Authors:** Yuling Meng, Qiang Zhang, Meixiang Zhang, Biao Gu, Guiyan Huang, Qinhu Wang, Weixing Shan

**Affiliations:** ^1^College of Plant Protection, Northwest A&F UniversityYangling, China; ^2^State Key Laboratory of Crop Stress Biology for Arid Areas, Northwest A&F UniversityYangling, China; ^3^College of Life Sciences, Northwest A&F UniversityYangling, China

**Keywords:** *Phytophthora parasitica*, protein disulfide isomerase, cell death, haustoria, plant infection, virulence factor

## Abstract

Protein disulfide isomerase (PDI) is a ubiquitous and multifunction enzyme belonging to the thioredoxin (TRX) superfamily, which can reduce, oxidize, and catalyze dithiol–disulfide exchange reactions. Other than performing housekeeping functions in helping to maintain proteins in a more stable conformation, there is some evidence to indicate that PDI is involved in pathogen infection processes. In a high-throughput screening for necrosis-inducing factors by *Agrobacterium tumefaciens*-mediated transient expression assay, a typical *PDI* gene from *Phytophthora parasitica* (*PpPDI1*) was identified and confirmed to induce strong cell death in *Nicotiana benthamiana* leaves. PpPDI1 is conserved in eukaryotes but predicted to be a secreted protein. Deletion mutant analyses showed that the first CGHC motif in the active domain of PpPDI1 is essential for inducing cell death. Using *P. parasitica* transformation method, the silencing efficiency was found to be very low, suggesting that *PpPDI1* is essential for the pathogen. Translational fusion to the enhanced green fluorescent protein (EGFP) in stable *P. parasitica* transformants showed that PpPDI1 is associated with haustoria-like structures during pathogen infection. Furthermore, the *PpPDI1-EGFP*-expressing transformants increase the number of haustoria-like structures and exhibit enhanced virulence to *N. benthamiana*. These results indicate that PpPDI1 might be a virulence factor of *P. parasitica* and contributes to plant infection.

## Introduction

Disulfide bonds, which are covalent linkages formed between the side chains of cysteine residues, play important roles for the stability of correct folding state of many proteins (Hatahet and Ruddock, 2009). The formation of disulfide bond is a critical step in the folding of nascent peptides in the endoplasmic reticulum (ER). In this process, increasing evidence supports the catalytic role of protein disulfide isomerase (PDI) family (Ellgaard and Ruddock, 2005; Appenzeller-Herzog and Ellgaard, 2008). The PDI family includes PDI and PDI-like proteins with thioredoxin domains, which varies in size, expression, localization and enzymatic function. In *Saccharomyces cerevisiae*, five PDI family members have been identified (Xiao et al., 2004), and in *Homo sapiens*, this number has increased to at least 21 members (Galligan and Petersen, 2012). Of the PDI gene family, the typical PDI is a multifunctional enzyme that fulfills key roles as ER foldases by catalyzing disulfide reduction (breakage), formation (oxidation), and isomerization (rearrangement), thereby promoting native protein folding (Tu et al., 2000; Galligan and Petersen, 2012).

A typical PDI consists of four domains that include two thioredoxin-like catalytic domains (a and a′) separated by two non-catalytic domains (b and b′). In addition to this, an ER retention signal is located at the small C-terminal domain (c), and also PDI has an N-terminal signal sequence. The two catalytic domains containing characteristic CGHC active-site motif are essential for PDI enzymatic activity (Appenzeller-Herzog and Ellgaard, 2008). While the non-catalytic domain “b′” provides the primary peptide or non-native protein-binding site, the other non-catalytic domain “b” may have a structural role in PDI rather than a direct catalytic role or binding role (Klappa et al., 1998; Ellgaard and Ruddock, 2005). However, ER sequences may differ greatly in amino acid composition, and also some PDI gene members do not contain this sequence (Galligan and Petersen, 2012). The domain composition has been known for years and the crystal structure for Pdi1p from *S. cerevisiae* was solved (Tian et al., 2006).

The classical PDIs, which possess an N-terminal signal peptide and a C-terminal ER retention signal, are abundant and normally retained in the ER and traditionally regarded as ER enzymes involved in protein folding. It is also discovered that PDI family members can undergo both co-translational import into the ER/secretory pathway and trafficking to compartments outside of the secretory pathway (Turano et al., 2002; Porter et al., 2015). The phenomenon of dual localization occurs in different species including mammalian (Turano et al., 2011), *Chlamydomonas reinhardtii* (Levitan et al., 2005), and *Arabidopsis thaliana* (Cho et al., 2011). In addition, there is convincing evidence showing that the host cell surface PDI-mediated disulfide bond reduction is involved in the infectious entry of a number of viruses (Gilbert et al., 2006; Ou and Silver, 2006; Jain et al., 2007) and bacteria (Abromaitis and Stephens, 2009). Indeed, *Toxoplasma gondii* PDI was identified in host tears, suggesting an extracellular location of infection (Meek et al., 2002). The mechanisms of dual trafficking of proteins within and outside the secretory pathway are discussed (Porter et al., 2015).

PDIs of pathogens play an important virulence role during host infection (Stolf et al., 2011). Increased expression of *Leishmania major PDI* (*LmPDI*) is correlated to virulence of the parasitic strains of *Leishmania* species that cause leishmaniases, suggesting that PDI protein is a virulence factor (Ben Achour et al., 2002). Using LmPDI as antigens to generate a vaccine, the BALB/c mice were partially protected against the pathogen (Benhnini et al., 2009). In *L. amazonensis*, PDI inhibitors can affect the parasite growth (Hong and Soong, 2008). *PfPDI-8* from *Plasmodium falciparum*, the most virulent malaria parasites, is expressed during all stages of parasite life cycle, and biochemical analysis revealed its role in facilitated folding of EBA-175, a leading malaria vaccine candidate (Mahajan et al., 2006).

*Phytopthora parasitica* is an oomycete plant pathogen with broad-range of host plants, best known for the black shank disease of tobaccos, and emerges as a model for oomycete pathogens (Meng et al., 2014). *P. parasitica* and its interaction with host plants, tobacco (Benhamou and Côté, 1992; Bottin et al., 1999), tomato (Kebdani et al., 2010), and *Arabidopsis* (Attard et al., 2010; Wang et al., 2011) have been characterized. Recently, draft genome sequences became available (http://www.broadinstitute.org/annotation/genome/Phytophthora_parasitica/MultiHome.html), which will accelerate the identification of genes that determine the molecular dialog between the species and host plants. Several pathogenicity factors have been identified in the species, including the elicitin-like ParA1 (Kamoun et al., 1993), NEP1-Like protein NPP1 (Fellbrich et al., 2002), and the apoplastic CBEL effectors (Mateos et al., 1997; Khatib et al., 2004), which are assumed to be perceived by the host plant cell surface receptors. Recently, PSE1, a RXLR effector of *P. parasitica*, was proved to favor pathogen infection by modulating auxin accumulation during the penetration process. However, the information of existing repertoire of *P. parasitica* molecules that are known to elicit plant defense responses or cell death has been limited.

We employed a high-throughput *Agrobacterium*-mediated transient expression assay to identify pathogenicity factors of *P. parasitica* that induce cell death on *Nicotiana benthamiana* and *N. tobacum* leaves. In the functional screening, we identified a typical *PDI* gene of *P. parasitica* (*PpPDI1*), which is active in causing strong necrosis on *N. benthamiana* leaves. Alignment of PDI sequences from different species revealed high level of conservation in the active domain in eukaryotic organisms. The necrosis-inducing activities, expression pattern, gene silencing, and over-expression analyses indicate that *PpPDI1* might play as a virulence factor contributing to *P. parasitica* infection and is likely essential for cell survival.

## Materials and methods

### Sequence analysis

The PDI protein sequences from different organisms were obtained from the National Center for Biotechnology Information (NCBI) (http://www.ncbi.nlm.nih.gov/), as following: *Phytophthora parasitica* (XP_008914616.1), *Phytophthora infestans* (XP_002895135.1), *Phytophthora sojae* (XP_009520350.1), *Saprolegnia parasitica* (KDO30563.1), *Albugo laibachii* (CCA26649.1), *Mus musculus* (NP_035162.1), *Homo sapiens* (NP_000909.2), *Arabidopsis thaliana* (NP_851234.1), *Triticum aestivum* (BAO79451.1), *Saccharomyces cerevisiae* (NP_009887.1), *Magnaporthe oryzae* (XP_003710672.1), *Leishmania major* (AAN75008.1), *Trypanosoma cruzi* (XP_821173.1), *Plasmodium falciparum* (CAC15387.1), *Toxoplasma gondii* (XP_002371293.1), and *Chlamydomonas reinhardtii* (XP_001701755.1). Alignments were generated using Clustal X. The conserved domain searches were performed using the NCBI CD search program (http://www.ncbi.nlm.nih.gov/Structure/cdd/wrpsb.cgi).

### *Phytophthora parasitica* culture conditions

The *P. parasitica* strains and transformants were routinely cultured on 5% (v/v) cleared carrot juice agar (CA) medium supplemented with 0.002% (w/v) β-sitosterol and 0.01% (w/v) CaCO_3_ in the dark at 23°C.

### Vector construction and *P. parasitica* transformation

To generate the silencing construct pTHS, a 291 bp fragment within *PpPDI1* coding region and a 319 bp fragment of *GFP* were amplified using primers listed in Table S1. The fragment of *PpPDI1* was digested with *Spe*I and *Cla*I, and ligated into a *Cla*I-digested kanamycin resistance gene linker, and the hairpin structure was then inserted into *Spe*I-linearized pBluescript II KS. To generate the co-silencing hairpin structure, the *GFP* fragment was digested with *Spe*I and *BamH*I, and ligated into the PpPDI1-linker-PpPDI1 fragment released from pBluescript II KS using *Spe*I. The co-silencing structure was then inserted into *BamH*I-linearized pBluescript II KS. At last, the GFP-PpPDI1-linker-PpPDI1-GFP fragment was released from pBluescript II KS using *BamH*I and blunt-ended by *Pfu* DNA Polymerase (Fermentas, USA), and then ligated into *Sma*I-linearized expression plasmid pTH210 (Judelson et al., 1991).

To generate the overexpression construct, the full-length *PpPDI1* ORF was amplified using PrimeStar polymerase (TaKaRa, China) from cDNA and was fused at C-termini to the *EGFP*.

The *P. parasitica* transformation was carried out using the polyethylene glycol–CaCl_2_ method as described (Bottin et al., 1999; Zhang et al., 2012). The *PpPDI1*-silencing construct was co-transformed into *P. parasitica* transformant 1121, which stably expresses GFP, with pTH209. The overexpression construct was co-transformed into strain Pp016 with pTH210. Transformants were recovered 3–7 days after regeneration on 5% CA, and the primary transformants were transferred to 5% CA with 4 μg/mL Geneticin and 80 μg/mL Hygromycin, respectively.

### RNA isolation and real-time RT-PCR analyses

To monitor *PpPDI1* expression level in *P. parasitica* by real-time RT-PCR, total RNA from vegetative hyphae (VH), sporulating hyphae (SH), cysts (CY), germinating cysts (GC), and infected plant tissues were extracted using TRIzol reagent (Invitrogen, USA) according to the manufacturer's protocol. For each samples, three biological replicates were performed. The first strand cDNA was synthesized from 0.5 μg total RNA, which was treated with gDNA Eraser and reverse transcribed using PrimeScriptRT reagent Kit (TaKaRa, China). Real-time RT-PCR experiments were carried out using SYBR Premix Ex TaqTM II (TaKaRa, China) according to the manufacturer's instructions. Primers used for real-time RT-PCR are described in Table S1. *WS041* (GenBank accession number CF891677), constitutively expressed throughout *P. parasitica* lifecycle stages, was selected as a normalizing reference gene (Shan et al., 2004). Relative levels of *PpPDI1* transcripts in *P. parasitica* were quantitated using the iQ5 real-time RT-PCR detection system (BioRad, USA).

### Pathogenicity assays

Detached leaves from 6-week-old *N. benthamiana* plants were maintained on moist filter paper in a plastic tray and were inoculated with 5% CA agar plugs grown with fresh *P. parasitica* mycelia. Total DNA was isolated from *P. parasitica*-infected tissues 60 h post inoculation (hpi). Real-time PCR was used to quantitate the colonization of *P. parasitica* to *N. benthamiana*, using primers specific for the constitutive genes of *N. benthamiana* and *P. parasitica* (Table S1). Three independent biological replicates were conducted.

### Microscopic examination

To visualize the *P. parasitica* structures, the samples were collected and viewed under Olympus BX-51 microscope equipped with differential interference contrast (DIC) optics. For microscopic characterization of the GFP signal of *P. parasitica* transformants, the examples were collected and viewed under Olympus BX-51 fluorescent microscope with the GFP filter (BP450-480).

To view infection and colonization of *P. parasitica* transformants in *N. benthamiana*, leaf samples were mounted in water and analyzed under a Nikon A1R^+^ confocal microscope using the wavelength of 488 nm for GFP signals.

### *Agrobacterium tumefaciens*-mediated transient expression assays

For *in planta* transient expression, *A. tumefaciens* strain AGL1 was used to deliver T-DNA constructs into 6-week-old *N. benthamiana* leaves. *A. tumefaciens* cultures with respective constructs were harvested and suspended in the infiltration medium [10 mM MgCl_2_, 10 mM 2-(N-morpholine)-ethane sulfonic acid (MES), pH 5.6, and 200 mM acetosyringone] to an OD_600_ = 0.4 prior to infiltration. The experiments were performed on at least six leaves. The *N. benthamiana* plants were grown and maintained throughout the experiments in a cultivation room with temperature of 22–25°C and high light intensity. Photographs were taken 3–5 days after infiltration.

## Results

### PpPDI1 induces strong necrotic response in *N. benthamiana*

**Figure 1:**
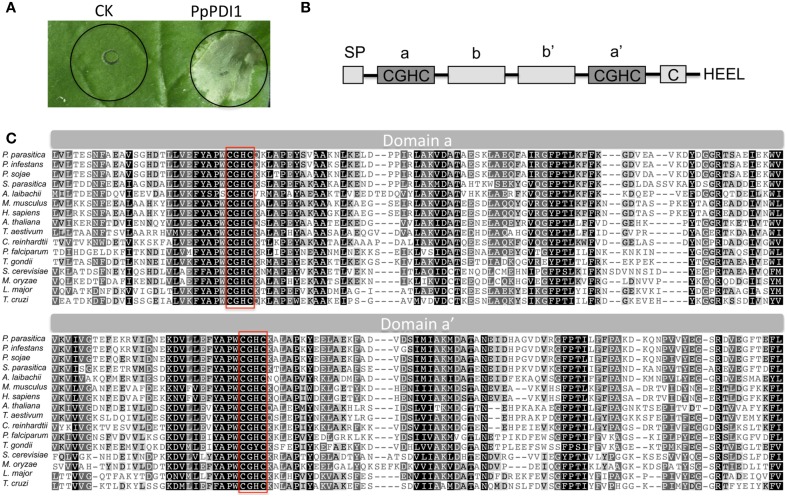
**Transient expression of ***P. parasitica PpPDI1*** induces strong cell death on ***N. benthamiana***. (A)** Infiltration of *Agrobacterium tumefaciens* strain AGL1 carrying *PpPDI1* construct into leaves of 6-week-old *N. benthamiana* plants, compared with the control construct GFP. The necrotic responses were scored and photographs were taken 3 days post infiltration (dpi). **(B)** Functional domains of PpPDI1 protein. PpPDI1 shows four TRX domains and two catalytic domains containing characteristic CGHC active-site motif. **(C)** Alignment of the active domains (a and a′) of *P. parasitica* PpPDI1 with PDI proteins of different organisms using the Clustal X program. Identical amino acid residues are highlighted against black background shading; similar amino acid residues are shaded with a light gray background, and distant similar amino acids are not shaded. The sequences for active motifs of CGHC are boxed.

Transient expression-based functional screening is a useful strategy for identifying protein factors important in plant–pathogen interaction. To search for pathogenicity factors that induce necrotic response, we constructed a cDNA library of infected tobacco tissues with *P. parasitica* strains using Gateway technology and performed a high-throughput *Agrobacterium*-mediated transient *in planta* expression assay on *N. benthamiana* and *N. tobacum*. This led to the identification of several cDNAs, including 1-B-10-8 that encodes a typical secreted PDI of *P. parasitica* (PpPDI1). Further overexpression experiments confirmed that PpPDI1 induces strong cell death on *N. benthamiana* leaves 3 days after infiltration (Figure 1A).

### PpPDI1 is conserved in eukaryotic species

PpPDI1 is comprised of 521 amino acids, including a signal peptide of 23 amino acid residues, four TRX domains with two catalytic domains (Cys-Gly-His-Cys), and a C-terminal ER retention sequence (His-Glu-Glu-Leu), as outlined in Figure 1B. *P. parasitica* genome encodes one copy of the classic PDI. Alignment of the amino acid sequences of PDIs from different species revealed that PDI is conserved at the active domain in eukaryotic organisms including some protozoans (Figure 1C). The PDI protein sequences are highly conserved in *Phytophthora* pathogens. For example, *P. parasitica* PDI was nearly identical to that of *P. infestans* (95%) and *P. sojae* (91%).

**Figure 2:**
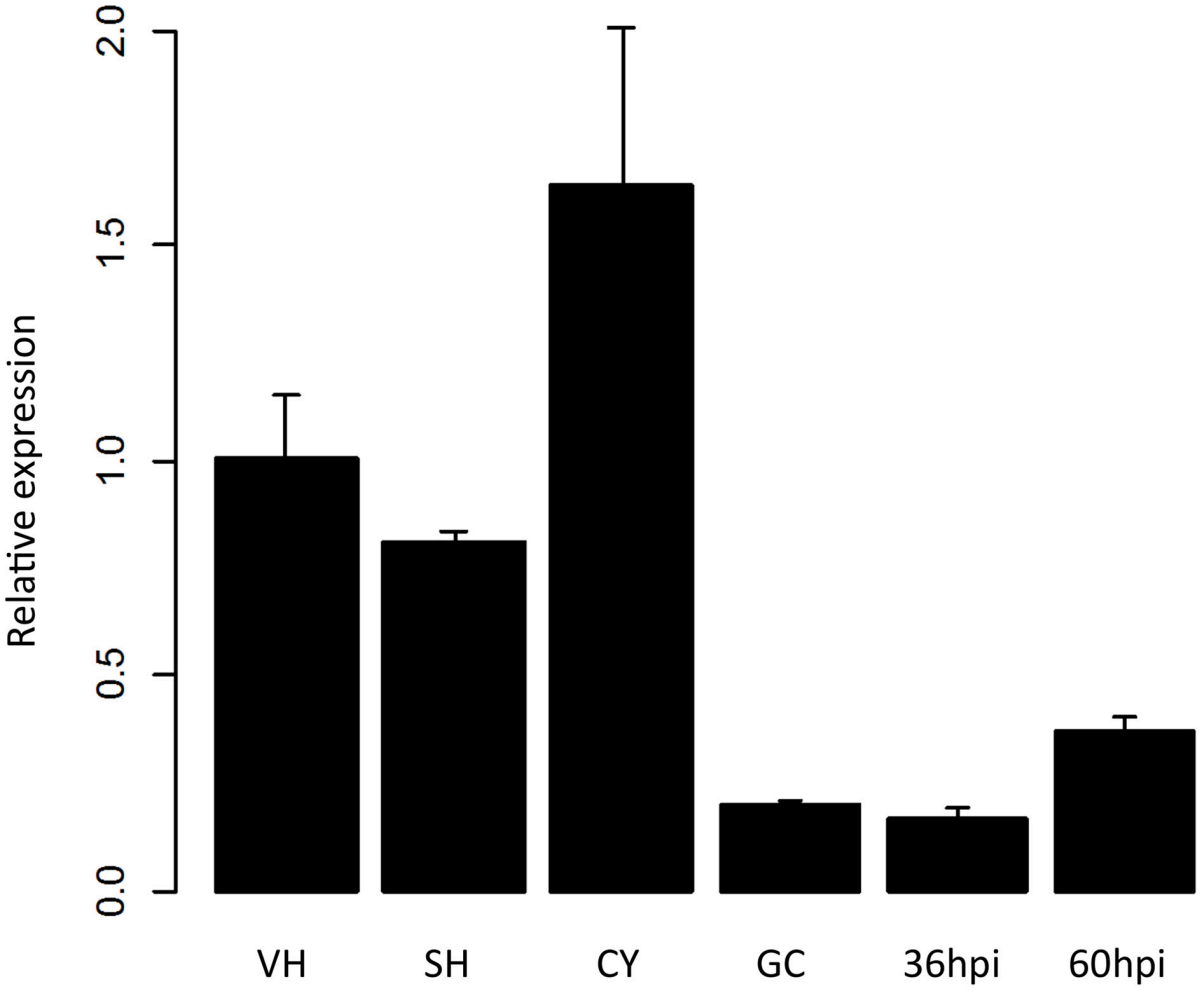
*****PpPDI1*** expression of ***P. parasitica*** during different developmental and infection stages**. Real-time RT-PCR was used to examine *PpPDI1* expression levels from vegetative hyphae (VH), sporulating hyphae (SH), cysts (CY), germinating cysts (GC), and infected stages at 36 and 60 hpi. *PpPDI1* expression levels are relative to that of the constitutively expressed *P. parasitica* gene *WS041*. Bars represent the standard errors of three biological replicates.

To explore whether the PpPDI1 is involved in plant infection, real-time RT-PCR was employed to analyze the expression of *PpPDI1* during distinct asexual development and infection stages. The results showed that *PpPDI1* is highly expressed in stages of vegetative hyphae (VH), sporulating hyphae (SH), and cysts (CY). *PpPDI1* is up-regulated during the late infection stage (60 hpi) compared to that in the germinating cyst (GC) and the early infection stages (36 hpi) (Figure 2).

### Functional domains of PpPDI1 required for cell necrotic induction

**Figure 3:**
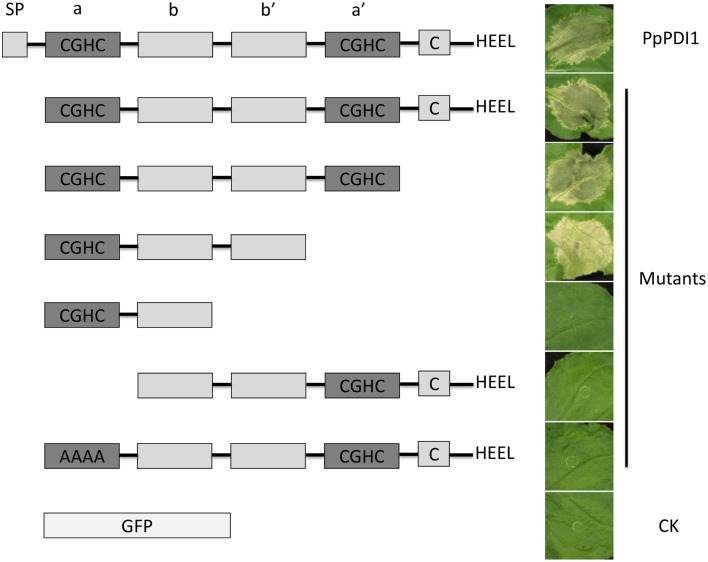
**Determination of domains of PpPDI1 required for cell death induction**. *In planta* transient expression of various N-terminal or C-terminal PpPDI1 deletion constructs to determine domains necessary for cell death induction. *A. tumefaciens* strain AGL1 cultures carrying different constructs were infiltrated into 6-week-old *N. benthamiana* leaves. The experiments were performed on leaves of at least six *N. benthamiana* leaves. The necrotic responses were scored and photographs were taken 3 days post infiltration (dpi).

To further define the domain of PpPDI1 required for cell death induction, deletion mutants were constructed and analyzed by *A. tumefaciens*-mediated transient expression in *N. benthamiana* (Figure 3). The results showed that the C-terminal deletion mutants with only a and b domains lost cell death-inducing activities, indicating that an intact C-terminus containing domains a, b, and b′ is required for full activity. The N-terminal deletion mutants without the first catalytic domain (a) lost cell death-inducing activities. The mutant, with the first CGHC motif replaced with alanine residues AAAA, lost the ability to induce cell death, indicating that the first active motif CGHC is essential for cell death induction of PpPDI1. These results showed that the region required for cell death induction includes the first catalytic domain (a) and the two domains (b and b′) with no known active motifs.

### PpPDI1 is likely essential for *P. parasitica*

**Figure 4:**
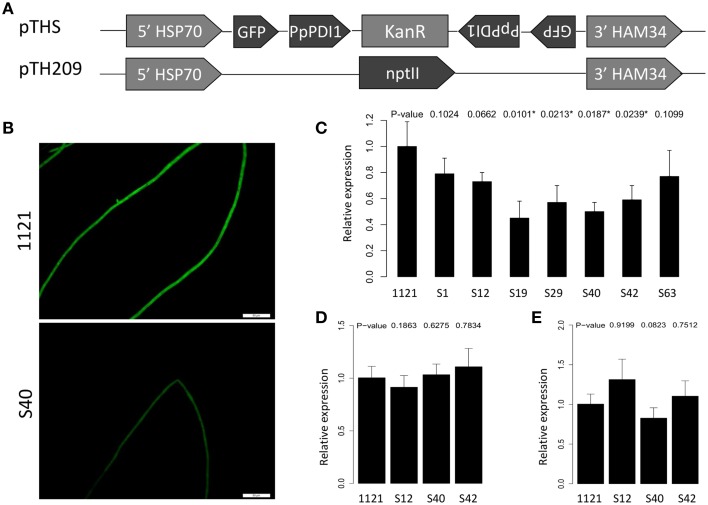
**Generation and analysis of ***PpPDI1***-silencing ***P. parasitica*** transformants. (A)** Constructs used for silencing in *P. parasitica* transformant 1121 that stably expresses GFP. In all constructs, transgenes are driven by the constitutive *Bremia lactuca HSP70* promotor and *HAM34* terminator. Plasmid pTHS contains 291 bp of *PpPDI1* and 319 bp of *GFP* in the sense and antisense orientations separated by the linker sequence [kanamycin-resistant gene (kanR)]. Plasmid TH209 contains the *nptII* gene and was used as the selection marker in the co-transformation of *P. parasitica*. **(B)** Hyphae of *PpPDI1*-silencing transformants (S40), showing significantly reduced GFP signal compared with the recipient transformant 1121 (bar, 50 μm). **(C)** Expression analysis of *PpPDI1* in the transformants with reduced GFP signals. Real-time RT-PCR evaluation of *PpPDI1* expression used hyphal RNA from the recipient transformant 1121 and seven transformants with significantly reduced GFP signals. Three transformants (S12, S40, and S42) were selected for further characterization. **(D)** Expression analysis of *PpPDI1* in *P. parasitica* 1121 and three transformants (S12, S40, and S42) during infection of *N. benthamiana*. Real-time RT-PCR evaluation of *PpPDI1* expression used hyphal RNA from the infection tissues 60 hpi. **(E)** Expression analysis of *PpPDI1* in *P. parasitica* 1121 and three transformants (S12, S40, and S42) subcultured for 3 weeks. Real-time RT-PCR evaluation of *PpPDI1* expression used hyphal RNA from the vegetative hyphae. All the real-time RT-PCR experiments were repeated three times with independent RNA isolations. Bars represent the standard errors of three biological replicates. *P. parasitica WS041* was used as a reference gene for quantification and normalization of *PpPDI1* expression.

To examine the function of *PpPDI1, P. parasitica* transformation was carried out to silence *PpPDI1*. To improve efficiencies in obtaining gene silencing *P. parasitica* transformants, we used *P. parasitica* transformant 1121 that stably expressed green fluorescent protein (GFP) as recipient to transform with plasmid pTHS (Figure 4A) containing a single construct to co-silence both *PpPDI1* and *GFP* genes. GFP signal intensities are, therefore, indicative of the extent of *PpPDI1* silencing. Plasmid TH209 carrying the *nptII* gene was used as the selection marker in the co-transformation of *P. parasitica*. In total, over 80 independent transformants were obtained, from which seven exhibited significantly reduced GFP signals (Figure 4B). Real-time RT-PCR analyses showed that *PpPDI1* expression was reduced in four of the seven transformants with up to 40–60% reduction compared to that of the recipient transformant 1121 (Figure 4C). Three transformants (S12, S40, and S42) including two silencing transformants were selected for further characterization.

To analyze the potential virulence role of *PpPDI1* in *P. parasitica*, the selected transformants, together with the recipient transformant 1121, were examined on *N. benthamiana* leaves. The infection lesions were measured for up to 60 hpi. However, the results showed that the *PpPDI1*-silencing transformants were similarly virulent to the recipient transformant 1121. Further analyses for the *PpPDI1* expression levels in the infection (Figure 4D) and the vegetative stages (Figure 4E) showed that *PpPDI1* expression was recovered in the *PpPDI1*-silencing transformants, though the GFP signals stayed weak compared with the recipient transformant 1121. The low level of silencing efficiencies and the recovered expression of *PpPDI1* in the silencing transformants indicated that *PpPDI1* expression is under strong selection, thereby suggesting an essential role it may play in *P. parasitica* development.

### *PpPDI1-EGFP*-expressing transformants produced finger-like structures

**Figure 5:**
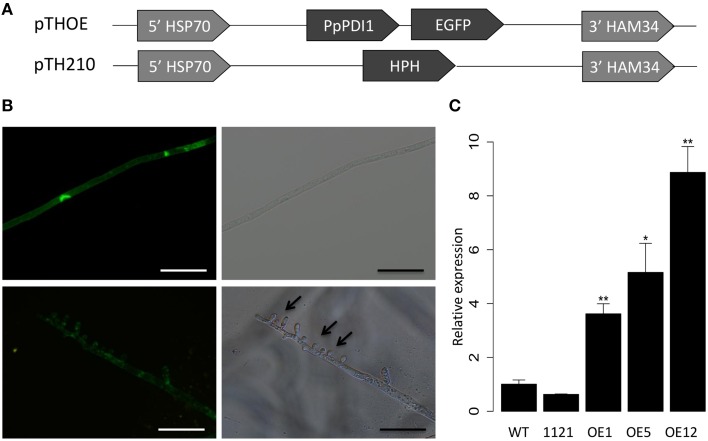
**Subcellular localization of PpPDI1 in ***P. parasitica***. (A)** Constructs used for expressing *PpPDI1-EGFP* in *P. parasitica* strain Pp016. In all constructs, transgenes are driven by the constitutive *Bremia lactuca HSP70* promotor and *HAM34* terminator. Plasmid pTHOE contains the full-length *PpPDI1* ORF fused at the C-terminus with the *EGFP*. Plasmid TH210 contains the *hygromycin*-resistance gene (HPH) and is used as the selection marker in the co-transformation of *P. parasitica*. **(B)** Cytological characterization of *P. parasitica* transformant OE5 expressing translational fusion of PpPDI1 with EGFP (bar, 50 μm). Arrows indicate finger-like structures. **(C)** Expression analysis of *PpPDI1* in transformants expressing *PpPDI1-EGFP*. Real-time RT-PCR evaluation of *PpPDI1* expression used hyphal RNA from the vegetative hyphae of the recipient strain Pp016, the control transformant 1121, and three transformants with GFP signals (OE1, OE5, and OE12). The real-time RT-PCR experiments were repeated three times with independent RNA isolations. Bars represent the standard errors of three biological replicates. *P. parasitica WS041* was used as a reference gene for quantification and normalization of *PpPDI1* expression. (^*^P < 0.05; ^**^P < 0.01).

To further examine the function of *PpPDI1, P. parasitica* transformants overexpressing *PpPDI1-EGFP* were generated (Figure 5A). In total, over 200 independent transformants were obtained and three transformants, OE1, OE5, and OE12, which exhibited stable GFP signals, were selected for further characterization. These three transformants showed normal colony morphology on 5% CA medium compared with the control with no obvious differences examined for 5 days. The GFP fluorescence signals were detected as irregular fluorescent spots (Figure 5B) in all the transformants. However, finger-like structures (Figure 5B) were frequently formed from hyphae at the colony edges. Real-time RT-PCR analyses confirmed that the *PpPDI1-EGFP* transcript levels were approximately increased 4-, 5-, and 9-fold, respectively, compared with the wild-type recipient strain Pp016 (Figure 5C).

### PpPDI1 is associated with haustoria-like structure during plant infection

**Figure 6:**
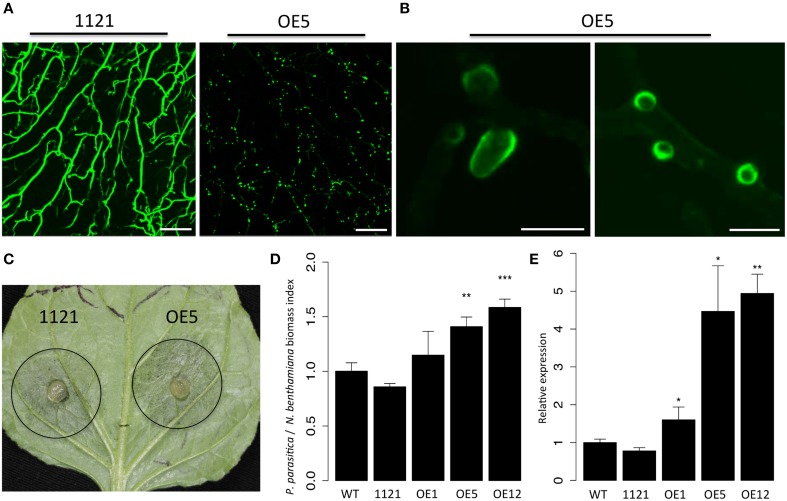
**PpPDI1 is associated with haustoria-like structures of ***P. parasitica*** during colonization of ***N. benthamiana***. (A)** Increased number of haustoria-like structures produced by *P. parasitica* transformant OE5 expressing PpPDI1-EGFP on infected *N. benthamiana* leaf tissue compared with the control transformant 1121 (bar, 100 μm). Confocal laser scanning microscopy was used to define hyphal cytoplasm and haustoria-like structures. **(B)** The PpPDI1-EGFP protein is highly enriched at periphery of the haustoria-like structures of *P. parasitica* during plant infection (bar, 10 μm). Confocal laser scanning microscopy was used to define the haustoria-like structures. **(C)** Leaves of 6-week-old *N. benthamiana* infected with *P. parasitica* transformant OE5 and the control transformant 1121. The leaves were inoculated with agar plugs containing *P. parasitica* mycelia. The photo was taken at 60 hpi. **(D)** Real-time PCR assay to determine *P. parasitica* biomass in infected plant tissues following inoculation with *P. parasitica*. Total genomic DNA from *P. parasitica* infected tissues was isolated at 60 hpi. Real-time PCR employed primers specific for the *N. benthamiana* and *P. parasitica* constitutively genes. Bars represent standard errors from three biological replicates. **(E)** Real-time RT-PCR assay is used to quantitate *PpPDI1* expression in *P. parasitica* wild-type recipient strain Pp016, the control transformant 1121, and three transformants expressing *PpPDI1-EGFP* (OE1, OE5, and OE12) during infection of *N. benthamiana*. The infection tissues were sampled 60 hpi. The real-time RT-PCR experiments were repeated three times with independent RNA isolations. Bars represent standard errors from three biological replicates. *P. parasitica WS041* was used as a reference gene for quantification and normalization of *PpPDI1* expression. (^*^P < 0.05; ^**^P < 0.01; ^***^P < 0.001).

Localization of a protein is implicative to its biological function and underlying mechanisms. To determine subcellular localization of PpPDI1 during *P. parasitica* infection, *N. benthamiana* leaves were inoculated with transformants OE1, OE5, and OE12 that express *PpPDI1-EGFP*. The result showed that, compared with the control transformant 1121, the *PpPDI1-EGFP*-expressing transformants produced more haustoria-like structures (Figure 6A, Figure S1A). Strikingly, the fluorescent signals were highly enriched at periphery of haustoria-like structures during plant infection (Figure 6B, Figure S1B). In contrast, the fluorescent signal of the control transformant 1121 is evenly distributed.

Pathogenicity assays on *N. benthamiana* leaves showed that the *PpPDI1-EGFP*-expressing transformants (OE1, OE5, and OE12) caused similar lesion sizes compared with the wild-type recipient strain Pp016 and the control transformant 1121 (Figure 6C). However, many more haustoria-like structures formed for the *PpPDI1-EGFP*-expressing transformants. Then real-time PCR analyses were performed to determine the *P. parasitica* biomass in the infected plant tissues. The results confirmed more colonization of *N. benthamiana* by the *PpPDI1-EGFP*-expressing transformants (Figure 6D) and the increased *PpPDI1* expression levels in the transformants during infection (Figure 6E). These results indicated that *PpPDI1* plays a positive role in the plant infection by *P. parasitica*.

## Discussion

In this study, we identified *PpPDI1* encoding a typical PDI in the oomycete pathogen *P. parasitica*. PpPDI1 induces strong necrosis in *N. benthamiana*. Translational fusion of *PpPDI1* with *EGFP* in *P. parasitica* transformants showed its association with the haustoria-like structures during infection of *N. benthamiana*. Transformants overexpressing *PpPDI1-EGFP* increased the number of haustoria-like structures and enhanced the virulence of the pathogen on *N. benthamiana*. These results indicated that PpPDI1 might play a virulence role in *P. parasitica*.

In the yeast, PDI has an essential function for cell survival, as its oxidase activity is critical to yeast growth and viability (Lamantia et al., 1991; Solovyov et al., 2004). PDI is also necessary for cell survival in mice since its deletion caused embryonic lethality (Hatahet and Ruddock, 2009). In our silencing experiments, the obtained transformants were poorly silenced with *PpPDI1*, with the highest silencing efficiency of 40%. Furthermore, the obtained *PpPDI1*-silencing transformants were shown to be reversed with the expression level of *PpPDI1* after subculturing. This was unlikely due to silencing transformation since the co-silencing of *GFP* was efficient. These results indicate that *PpPDI1* is likely an essential gene for *P. parasitica*.

Being a typical PDI protein, PpPDI1 contains the main structural building block. The active domain of PpPDI1 is highly conserved within eukaryotic organisms. The a-type domains contain two cysteines in a CXXC active-site motif with an intervening GH sequence, which is the most common CGHC motif in the PDIs. PpPDI1 is capable of triggering cell death on *N. benthamiana*. The region required for cell death induction includes the first domain (a) with active motif (CGHC) and the two non-active domains (b and b′). However, the mutant, with domains a–b–b′, caused cell death at a reduced level (Figure 3). The mutant with the active motif CGHC being replaced with alanine residues AAAA abolished necrosis-inducing activity, indicating that the cell death-inducing function might be related to the catalytic properties.

Host cell death is a common feature of most plant–pathogen interactions. However, the timing and control of cell death play different roles in the outcome of parasitic interaction. *Phytophthora* species, the hemibiotrophic oomycetes, initiate host cell death during a later necrotrophic stage of infection (Catanzariti et al., 2007). In the initial biotrophic phase, the pathogen proliferates asymptomatically, requiring the living cells in the host with efficient mechanisms to evade and suppress host defenses. In the second stage, there appears large-scale cell death and tissue dissolution, which mediated by different strategies such as secretion of lytic enzymes and cell-death elicitors, resulting in profuse colonization and sporulation (Kelley et al., 2010). Some cell death-inducing proteins in *Phytophthora* species have been reported. For example, Nep1-like proteins (NLPs), which induce defense responses in both susceptible and resistant plants, are broadly distributed in *Phytophthora* (Fellbrich et al., 2002; Qutob et al., 2002; Kanneganti et al., 2006). In *P. sojae* and *P. infestans*, the *NLP* genes are expressed at late stage of host infection and may facilitate colonization of host tissues during the necrotrophic growth. Although the classical PDIs are highly abundant and normally retained in the ER, it is also present in the cytosol, nucleus, and on the cell surface (Turano et al., 2002). However, the mechanisms governing escape of PDI from the ER are not clear. In our study, PpPDI1 induces strong cell death in host plant *N. benthamiana*. *PpPDI1* is expressed in all stages but up-regulated during late infection stage in *P. parasitica* compared with germinating cyst (GC) stage and the early infection stage (36 hpi). These results suggest that other than performing housekeeping function in the ER, it may function in triggering host necrosis to facilitate pathogen colonization during the necrotrophic phase of infection.

Filamentous eukaryotic plant pathogenic microorganisms such as the obligate biotrophic fungi and oomycetes frequently form the specialized infection structures called haustoria inside the cells. Haustoria play a major role in delivering nutrients such as sugars and amino acids from the host into biotrophic parasites (Hahn et al., 1997; Szabo and Bushnell, 2001; Voegele et al., 2001). There is also evidence to suggest involvement of haustoria in the redirection of host metabolism and the suppression of host defenses (Voegele and Mendgen, 2003) and in the transferring of effector proteins into host plant cells (Catanzariti et al., 2006, 2007; Whisson et al., 2007), suggesting an important role haustoria may play during plant infection. Under our inoculation conditions on *N. benthamiana*, abundant *P. parasitica* haustoria-like structures were produced at 48–60 hpi, and real-time RT-PCR assays showed that *PpPDI1* is up-regulated during the later biotrophic stage of infection (60 hpi), when necrotrophic stage starts, compared with germinating cyst (GC) and the earlier biotrophic infection stages (36 hpi). Furthermore, when compared with the irregular fluorescent spots (Figure 5B) cultured on 5% CA medium, the GFP fluorescent signal was highly enriched at periphery of the haustoria-like structures (Figure 6B) in *PpPDI1-EGFP*-expressing transformants during infection. In addition, overexpression of *PpPDI1-EGFP* in *P. parasitica* increased the number of haustoria-like structures and enhanced the virulence to *N. benthamiana*. These results indicate that PpPDI1 may play an important role in virulence, possibly by accelerating development of haustoria-like structures and necrotrophic stage of plant infection.

Several studies indicate that Dsb (disulfide bond) proteins, homologs of PDI in bacteria, play a crucial role in pathogenesis (Lasica and Jagusztyn-Krynicka, 2007). The most common oxidative folding catalyst DsbA affects survival and virulence of the pathogens, and is involved in the biogenesis of functional pili and secretion system in many bacterial species (Yu and Kroll, 1999; Lasica and Jagusztyn-Krynicka, 2007; Heras et al., 2009). As it does in bacteria, *PDI* expression is reported to positively correlate with the pathogenicity, especially the intracellular protozoans (Stolf et al., 2011). However, the underlying mechanisms are far less understood. In our study, we propose that PpPDI1 is a virulence factor contributing *P. parasitica* infection on *N. benthamiana*. First, PpPDI1 induces strong cell death on the host, which is consistent with its up-regulated expression in later biotrophic infection stages. It may play as a cell death factor to trigger host necrosis to facilitate pathogen colonization during the following necrotrophic phase of infection. Second, PpPDI1 is associated with the haustoria-like structures in the infected plant tissues. Furthermore, overexpression of *PpPDI1-EGFP* increased the number of haustoria-like structures and even increased the production of finger-like structures in the absence of host tissues. As a chaperon protein, PpPDI1 may facilitate correct folding of haustoria structural proteins or pathogenesis-related proteins to enhance the virulence of the pathogen on host plants. However, the actual localization of PpPDI1 and the role of PpPDI1 in *P. parasitica* biology and pathology remain to be determined.

## Author contributions

Conceived and designed the experiments: WS; Performed the experiments: YM, QZ, MZ, and BG; Analyzed the data: WS, YM, and QW; Contributed reagents/materials/analysis tools: YM, QZ, and GH; Wrote the paper: WS and YM, with contribution from all authors.

### Conflict of interest statement

The authors declare that the research was conducted in the absence of any commercial or financial relationships that could be construed as a potential conflict of interest.
