# Supplementary material for: The protein disulfide isomerase 1 of Phytophthora parasitica (PpPDI1) is associated with the haustoria-like structures and contributes to plant infection
Source: Front Plant Sci. 2015 Aug 18;6:632. doi: 10.3389/fpls.2015.00632 (PMC4539480; doi:10.3389/fpls.2015.00632)
Supplement: Supplementary file 1 [file DataSheet1.DOCX]

***Supporting Information***

**The protein disulfide isomerase 1 of *Phytophthora parasitica* (PpPDI1) is associated with the haustoria-like structures and contributes to plant infection**

**Yuling Meng^1,2^, Qiang Zhang^1,2^, Meixiang Zhang^3,#^, Biao Gu^1,2^, Guiyan Huang^2,3^, Qinhu Wang^2,3^, Weixing Shan^1,2^***

^1^ College of Plant Protection, Northwest A&F University, Yangling, Shaanxi, People’s Republic of China

^2^ State Key Laboratory of Crop Stress Biology for Arid Areas, Northwest A&F University, Yangling, Shaanxi, People’s Republic of China

^3^ College of Life Sciences, Northwest A&F University, Yangling, Shaanxi, People’s Republic of China

^#^Current Address: College of Plant Protection, Nanjing Agricultural University, Nanjing, Jiangsu, People’s Republic of China

*** Correspondence:** Weixing Shan, College of Plant Protection, Northwest A&F University, 3 Taicheng Road, Yangling, Shaanxi 712100, China.

email: wxshan@nwafu.edu.cn

**Figure S1 PpPDI1 is associated with *P. parasitica* haustoria-like structures during colonization of *N. benthamiana* in the epidermal cells. (A)** Increased number of haustoria-like structures produced by *P. parasitica* transformant OE5 expressing PpPDI1-EGFP in infected *N. benthamiana* leaves, compared with the control transformant 1121 (bar, 100 μm). **(B)** The PpPDI1-EGFP protein is highly enriched at periphery of the haustoria-like structures of *P. parasitica* during plant infection (bar, 10 μm). Arrows indicate haustoria-like structures.

**Table S1 Primers used in this study.**

(Restriction sites underlined)

| **Purpose** | **Primer** | **Sequence (5’ → 3’)** |
| --- | --- | --- |
| Construction of vector pTHS | PpPDI1_SpeI_F | CATGACTAGTGTGGCCTCCACCAGCGCTGAC |
|  | PpPDI1_ClaI_R | CATGATCGATGTTGGCCTTGGCCGGAGCCTTG |
|  | EGFP_BamHI_R | CATCGCGGATCCCACCCTCGTGACCACCTTC |
|  | EGFP_SpeI_R | TAGATCGGACTAGTCACCTTGATGCCGTTCTTCT |
| Construction of vector pTHOE | PpPDI1_SmaI_F | GATTCCCGGGATGATGTTCATGAAGCAG |
|  | pTHOE_R | GCTCACCATCAGCTCCTCGTGCTCGGC |
|  | pTHOE_F | GAGGAGCTGATGGTGAGCAAGGGCGAG |
|  | EGFP_SmaI_R | CTAACCCGGGTTACTTGTACAGCTCGTC |
| Real-time RT-PCR for *PpPDI1* expression | PpPDI1_F | TCTTCCCTGCCAAGGACAAAC |
|  | PpPDI1_R | TCTTCTCCTCCTGCTCCACCTC |
|  | WS041_F | CACGTACACATGCCCGAGAC |
|  | WS041_R | TTCCCATGTAGGCCGAGTATTC |
| Real-time PCR for DNA biomass | PpUBC_F | CCACTTAGAGCACGCTAGGA |
|  | PpUBC_R | TACCGACTGTCCTTCGTTCA |
|  | NbAct_F | ACCATCAATGATCGGAATGG |
|  | NbAct_R | GCTCATCCTATCAGCAATGC |
| Construction for PpPDI1 mutants | PpPDI1_SmaI_F | GATTCCCGGGATGATGTTCATGAAGCAG |
|  | PpPDI1_KpnI_R | CTAAGGTACCCTACAGCTCCTCGTGCTC |
|  | PpPDI1_M1_F | GATTCCCGGGATGGCTGAGTTCGAGGAGGAG |
|  | PpPDI1_M1_R | PpPDI1_KpnI_R |
|  | PpPDI1_M2_F | PpPDI1_M1_F |
|  | PpPDI1_M2_R | CTAAGGTACCCTACAGGAACTCCGTGAAGCC |
|  | PpPDI1_M3_F | PpPDI1_M1_F |
|  | PpPDI1_M3_R | CTAAGGTACCCTACTCGTCACTGTCGTCCTC |
|  | PpPDI1_M4_F | PpPDI1_M1_F |
|  | PpPDI1_M4_R | CTAAGGTACCCTACTCCTGCGAGAACGTGAT |
|  | PpPDI1_M5_F | GATTCCCGGGATGAAGAAGTCGGGCCCTGCTG |
|  | PpPDI1_M5_R | PpPDI1_KpnI_R |
|  | PpPDI1_M6_1F | PpPDI1_M1_F |
|  | PpPDI1_M6_1R | CTTCTGAGCTGCTGCAGCCCAAGGTGCGTAGAATTC |
|  | PpPDI1_M6_2F | GCTGCAGCAGCTCAGAAGCTGGCGCCTGAGTAC |
|  | PpPDI1_M6_2R | PpPDI1_KpnI_R |
