# Supplementary figures and images for: The protein disulfide isomerase 1 of Phytophthora parasitica (PpPDI1) is associated with the haustoria-like structures and contributes to plant infection
Source: Front Plant Sci. 2015 Aug 18;6:632. doi: 10.3389/fpls.2015.00632 (PMC4539480; doi:10.3389/fpls.2015.00632)

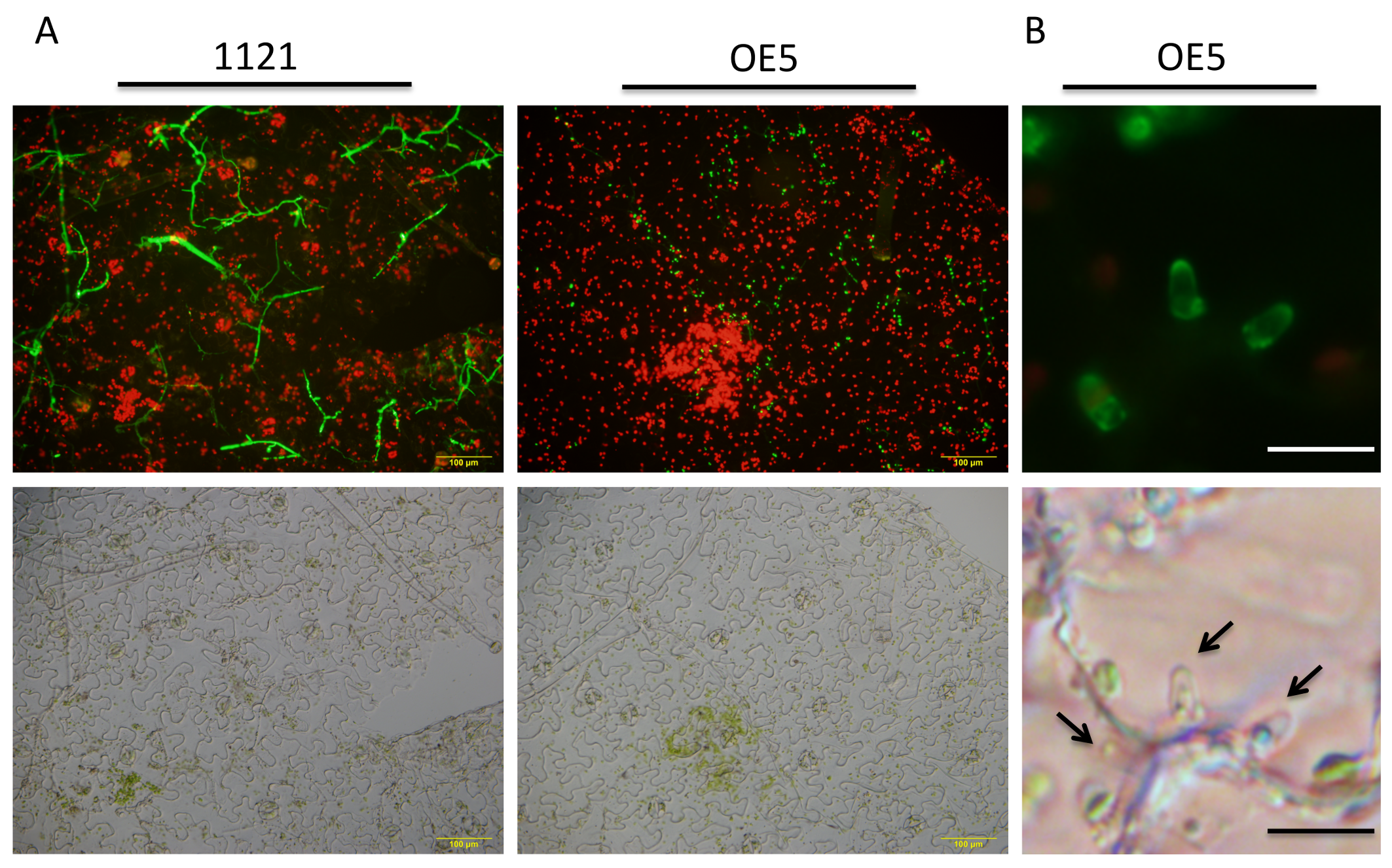

Supplement: Supplementary file 2 [file Image1.TIF]
